# Fabrication Quality Assessment Based on the Coupling of a Dual-Core Microstructured Polymer Optical Fiber

**DOI:** 10.3390/s21227435

**Published:** 2021-11-09

**Authors:** Amaia Berganza, Eneko Arrospide, Josu Amorebieta, Joseba Zubia, Gaizka Durana

**Affiliations:** 1 Department of Applied Mathematics, University of the Basque Country, 48013 Bilbao, Spain; eneko.arrospide@ehu.eus (E.A.); josu.amorebieta@ehu.eus (J.A.); 2 Department of Communications Engineering, University of the Basque Country, 48013 Bilbao, Spain; joseba.zubia@ehu.eus (J.Z.); gaizka.durana@ehu.eus (G.D.)

**Keywords:** microstructured polymer optical fiber, fabrication, dual-core fiber, coupler

## Abstract

In this paper we report on the theoretical analysis and fabrication of a dual-core microstructured polymer optical fiber (mPOF) and demonstrate how the coupling characteristics of a dual-core mPOF may be a key factor to assess the quality of the fabrication process. The coupling characteristics of this fiber have been tested and, for comparison purposes, simulations regarding the effects of inaccuracies during the manufacturing process were carried out to evaluate the fabrication quality. Results indicate that theoretical, simulation and experimental data are in good agreement, which highlights the uniformity of the microstructure along the fiber and the quality of its fabrication process. In fact, the manufactured mPOF reached a coupling efficiency up to 95.26%, which makes this mPOF appealing for applications in which highly efficient power couplers are required.

## 1. Introduction

In the last several years, interest in microstructured optical fibers (MOFs), or photonic crystal fibers (PCFs), has increased significantly as a consequence of their potential to be used in a large number of applications such as sensors [1,2,3,4], polarization splitters [5,6,7] and power splitters [8,9,10], among others. Briefly, these fibers consist of a periodic arrangement of holes in the micron and submicron scale with high refractive index contrast, which can guide the light along a solid or hollow core. While light in solid-core MOFs guides by total internal reflection as the holey cladding has a lower refractive index than the core [11,12]; in the case of hollow-core MOFs the light guiding mechanism along the core is based on the photonic bandgap effect, inhibited coupling or anti-resonance [11,13,14].

Commonly, the fabrication process of MOFs consists of three steps [11]: the first is manufacturing a preform, i.e., an enlarged version of the final fiber. The second step involves drawing the preform to obtain an intermediate cane, which allows greater manufacturing control over the drawing process, and the last step consists in both sleeving the aforementioned cane into a polymer tube and going through another drawing process in order to obtain the final fiber. Although there are several different MOF fabrication techniques such as molding, extrusion, 3D printing, etc. [11,15,16,17], in general, silica MOF preforms are fabricated using the capillary stacking technique where capillaries are manually stacked according to the desired pattern and, afterward the resulting structure is inserted into a jacketing tube. The main drawback of this technique is its limitation to triangularly packed capillary structures, as well as being time-consuming. As an interesting alternative, we can mention the preform fabrication technique, an easier and more versatile method that allows more complex microstructure patterns and automation. These facts make it suitable for research manufacturing. Although there are some drilling techniques for glass fiber preforms [18], when polymer preforms come into play, it represents one of the preferred fabrication methods. Furthermore, a heating strategy that guarantees uniform heating of the cross-section is of paramount importance if we want to keep the deformation of the microstructure to a minimum [19]. In comparison to their glass counterparts, polymer optical fibers (POFs) bring new opportunities in several fields of interest. Although they have higher signal attenuation, dispersion ratios and require specific connectors, features that make them unappealing for long-distance communication systems [20], they are lighter, more flexible, less expensive and easier to handle, and, in addition, they have a high sensitivity to strain and a much lower drawing temperature, making it possible to incorporate new dopants into them [21]. For all those reasons, POFs are excellent candidates for their use in many applications.

Multicore fiber (MCF) technology emerged as a candidate to overcome the data capacity limit of optical communication systems. However, they have also attracted much attention in the field of optical sensing [22,23]. Among the different design options offered by MCFs for sensing applications, dual-core (DC) design deserves special attention for its ability to operate in interferometric configurations [24]. The power coupling between cores may be used advantageously to measure strain [25], temperature [26] or refractive index [27], but also brings the opportunity to provide a tool for evaluating the fabrication quality of microstructured POFs (mPOFs).

In this paper, we demonstrate how the coupling characteristics of a dual-core mPOF can be used to assess the quality of the fabrication process of mPOFs. First, we report on the process of mPOF fabrication particularized to the case of a single-mode (SM) DC mPOF. Then, the theoretical aspects of interest of this type of fiber are presented, together with the numerical simulations that indicate the particular aspects to consider for assessing the fabrication quality of mPOFs. Afterward, the numerical and experimental results are presented, compared and discussed, to finish with the most relevant conclusions that support the research presented in this paper.

## 2. Materials and Methods

### 2.1. Materials

Amongst the different polymers used to fabricate MOFs, poly (Methyl methacrylate) (PMMA), polycarbonate (PC), fluorinated polymer CYTOP, cyclo-olefin homopolymer (ZEONEX), and cyclic olefin copolymers (TOPAS) are the most common, and each provide specific mechanical, chemical and physical properties to the final fiber. Among them, PMMA is the most usually employed for its low cost and high transparency in the visible wavelength region. In this work, we considered extruded PMMA rods and tubes commercialized by Evonik (Essen, Germany) for the drawing process of the preforms. The most significant properties of this material follow: coefficient of linear thermal expansion around 7–11 × 10^−5^ K^−1^, low thermal conductivity of 0.19 W m^−1^ K^−1^, water absorption of 23 mg (ISO 62, Method 1) and transmittance approximately 92% [28]. To obtain good processability of this material, it is necessary to consider the weight-average molecular mass. High values of this parameter imply that the polymer does not flow, even at high temperatures above its glass transition temperature (around 105 °C), instead showing a rubber-like behavior. Using the gel permeation chromatography technique, a value of around 170,000 g/mol was measured in our samples. Additionally, a thermal treatment is essential for a correct drawing as it eliminates volatile impurities such as unreacted monomer particles, the rest of initiators or solvent residues, and to remove the absorbed moisture [29,30]. To that end, the rods and tubes used in this work were annealed at 90 °C for two weeks.

### 2.2. Fabrication

As explained in the introduction, the fabrication process of MOFs consists of three steps: first, fabricating a structured preform; then drawing the preform to obtain an intermediate cane; and finally drawing the cane down to the final fiber.

The preform with the desired structure was fabricated using the drilling method. This method presents two fundamental aspects that must be considered initially. On the one hand, it is a time-consuming process, as the geometry of the structure could be composed of a large number of holes. During the drilling process, in order to remove the generated heat properly and avoid the harmful effects of polymer overheating, using conventional drill bits requires a continuous supply of coolant into the hole and a continuous extraction of the generated chips, extending the processing time far more than the time expected for conventional drill bits. On the other hand, as a result of using conventional drill bits, short structured preforms are obtained. In order to overcome these drawbacks, we used a cooling lubricant (a mixture of BIOSOL-CO oil and water) and a single-lip gundrill [31], 3 mm in diameter and a length-to-diameter ratio of 50. This drill bit has a kidney-shape inner channel through which the coolant enters. The coolant refrigerates the cutting edge and the periphery of the drill, and evacuates the chips as they are generated through an external V-shaped channel. This new drill bit design allows drilling each hole in one and continuous infeed with a considerable reduction of the drilling time [19]. In addition, the 50× length allows for achieving longer preforms. Furthermore, the drill bit includes guide pads that provide a self-guiding effect, thus improving the surface smoothness of the inner wall of the holes [32]. 

**Figure 1:**
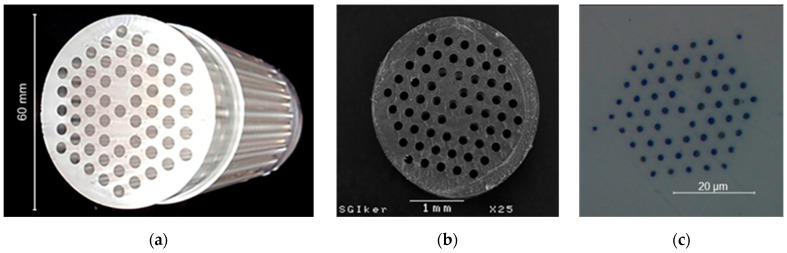
Photographs of (**a**) the fabricated PMMA preform, (**b**) the intermediate PMMA cane and (**c**) the cross-section of the final DC mMOF.

The structured preform was drilled in a computer numerical control (CNC) multiprocess machining center, Ibarmia ZVH 38-L1600 (Ibarmia, Azkoitia, Basque Country (Spain)). A 150 mm-long and 60 mm-diameter PMMA rod was fixed in a three-jaw chuck plate and the bulk rod was drilled through a programmed and automated sequence of orders. Four hexagonal rings were drilled on the PMMA rod. However, two defects were left in the first ring to operate as the two guiding cores of the final fiber. The drilled preform is shown in Figure 1a.

Afterward, the preform-to-fiber drawing was carried out using a two-step procedure. In the first step, the drilled preform was adequately heated and stretched down to an intermediate cane (see Figure 1b). In the second step, the intermediate cane was assembled into a sleeving tube and both were fused using vacuum during the final drawing to the DC fiber. Homogeneous radial and azimuthal distribution in the preform temperature was crucial during the heating, as nonuniform temperatures could modify the symmetry of the original structure and thus affect the coupling between cores. However, the poor thermal conductivity of PMMA complicates the homogeneous heating of the thick preform. In order to overcome this difficulty, the preform was first preheated in a radiative furnace using a step-like heating, and then moved down into a resistive furnace where the preform was stretched [30] to a 3 mm-diameter cane. The cane was next sleeved in a 20 mm-outer diameter tube and drawn to fiber using a feeding speed of 0.5 mm/min and a drawing speed of 3135 mm/min. During the drawing process and using a pressurizing device, a vacuum pressure of −150 mbar was applied to fuse the tube with the cane and positive pressure of 3.8 mbar to counter the contraction forces created during the drawing process, minimize hole deformation and achieve the desired diameter. The maximum temperature of the radiative furnace was 220 °C. The final fiber presented a 250 µm diameter, an average hole diameter of 1.7 µm and an average pitch of 4.1 µm, resulting in a ratio of hole diameter to pitch of 0.414, which represents the limit value for endlessly single-mode guidance [33]. Experimentally, the existence of a single-guided mode was evidenced by examining the far-field pattern, and observing that it was independent of the exact fiber location or state. The cross-section of the fiber is shown in Figure 1c. The small holes out of the four hexagonal rings are imperfections created during the fusion process of the sleeving.

### 2.3. Coupled-Mode Theory

**Figure 2:**
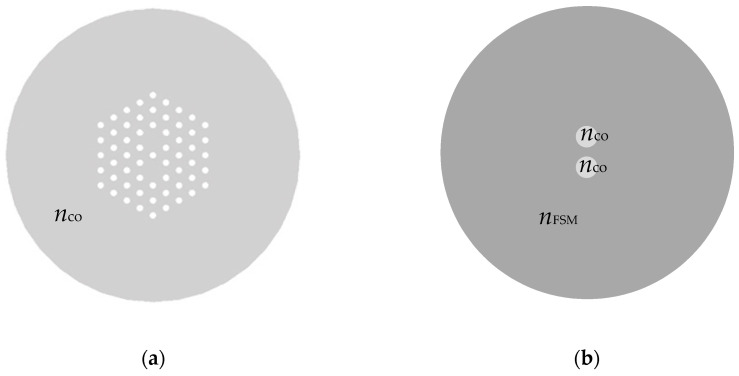
(**a**) Cross-section of (**a**) the simulated mPOF and (**b**) the equivalent dual-core step-index fiber.

The coupling process in a DC SM fiber is described as a special case of the coupled-mode theory [34]. Furthermore, we can consider the DC mPOF (shown in Figure 2a) as a conventional DC step-index fiber (shown in Figure 2b). In this approach, the equivalent fiber structure has two cores of refractive index *n*_co_, and the radius of the equivalent cores is chosen to be Λ/2, where Λ is the pitch, that is, the separation between the centers of two consecutive air holes in the microstructure. This resulting value of the radius is the best choice for the scalar analytical approach used in this section, as it is thoroughly explained in [35]. Additionally, within the cladding, the propagative solution to Maxwell’s equation with the largest *n*_eff_ is named the fundamental space-filling mode (FSM), whose effective refractive index is *n*_FSM_. That is, the FSM mode is the fundamental mode of the infinite microstructure when the cores are absent. This way, the cladding of the mPOF behaves as a homogeneous material of refractive index *n*_FSM_, such that guided modes propagate with an effective refractive index *n*_eff_ that fulfils the condition *n*_FSM_ < *n*_eff_ < *n*_co_ [35,36].

According to the theory, in the case of a DC SM mPOF, the fields in the fiber are well approximated by a linear combination of the fundamental-mode fields of each SM core in isolation. Coupling occurs as a consequence of the interference between those fields that cause an exchange of power between the cores as the light propagates along the fiber. More precisely, the power at each core is given by

(1) $$\begin{array}{c} P_{1}\left(z\right)={\left|b_{1}\left(z\right)\right|}^{2}=\cos^{2}\left(Cz\right)=1-\sin^{2}\left(Cz\right)\\ P_{2}\left(z\right)={\left|b_{2}\left(z\right)\right|}^{2}=\sin^{2}\left(Cz\right) \end{array}$$

where *b*_1_ (*z*) and *b*_2_ (*z*) are the SM field amplitudes along the longitudinal axis of the fiber (*z*), and *C* is the coupling coefficient between the two cores, which is a factor that describes the overlap of each fundamental mode in the neighboring core. From Equation (1), the coupling length may be determined, i.e., the distance for full power transfer from one core to the other:

(2) $$L_{c}=\frac{\pi }{2C},$$

where *C* is given by (assuming identical cores [34,37]):

(3) $$C=\frac{\sqrt{2\Delta }}{\rho }\frac{U^{2}}{V^{3}}\frac{K_{0}\left(2W\Lambda /\rho \right)}{K_{1}^{2}\left(W\right)}.$$

In this expression, *ρ* is the radius of each of the cores, *V* is the normalized frequency given by $V=k_{0}Rn_{co}\sqrt{2\Delta }=\sqrt{U^{2}+V^{2}}$, where $\Delta =\left(n_{co}^{2}-n_{FSM}^{2}\right)/\left(2n_{co}^{2}\right)$ is the relative refractive-index difference, and *U* and *W* are solutions of the characteristic equation

(4) $$U\;J_{1}\left(U\right)/J_{0}\left(U\right)=W\;K_{1}\left(W\right)/K_{0}\left(W\right).$$

*J*_0_ and *J*_1_ are the zeroth-order and first-order Bessel functions of the first kind, and *K*_0_ and *K*_1_ are the zeroth-order and first-order modified Bessel functions of the second kind, respectively. Solving this equation numerically, we can obtain both the effective refractive indices of the fundamental mode (*n*_eff_) and *n*_FSM_, whose values allow us to calculate the coupling length.

### 2.4. Numerical Simulations

As shown later, the results from the numerical simulations provide the reference performance that may be expected from a DC SM mPOF that followed the appropriate fabrication process. More precisely, we performed several numerical simulations on a DC mPOF in order to obtain the corresponding values of *n*_eff_ and *n*_FSM_, and from there determine the coupling characteristics of the aforementioned fiber. As shown in Figure 2a, the microstructure is composed of four hexagonal rings of air holes around two solid cores of PMMA (refractive index of 1.4937 at 532 nm). The values for the hole radii and pitch are chosen to match the average values experimentally measured in the fabricated mPOF (Section 2.2): ρ=0.85 μm and Λ=4.1 μm. In this case, the hole diameter-to-pitch ratio is 0.41, a value that guarantees the SM operation of each core necessary to apply the coupled-mode theory explained in Section 2.3 [33]. For the simulations, a wavelength of 532 nm was considered. The value of *n*_FSM_ was numerically determined from the infinite periodic cladding, i.e., in the absence of the two solid cores [36].

The numerical simulations also aim at allowing analysis of how certain structural imperfections on the fiber microstructure affect the coupling characteristics of the fiber. More specifically, the size of certain groups of holes is slightly increased with respect to the nominal values given above. The selected groups of holes, as well as their effect on the coupling characteristics, are presented in greater detail in Section 3.

The numerical modeling relies on the eigenmode expansion propagation solver of the Lumerical’s MODE simulator, which is based on a fully vectorial frequency domain method for solving Maxwell’s equations [38].

### 2.5. Experimental Set-Up

Several experimental set-ups were assembled for the experimental characterization of the fabricated mPOF, which ultimately led to the assessment of the fabrication process quality. We describe each to some extent.

#### 2.5.1. Coupling Length Measurement

**Figure 3:**
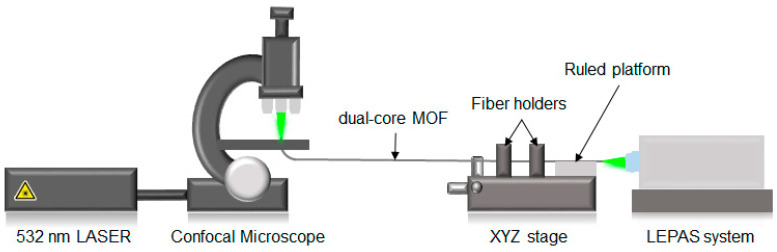
Schematic diagram of the experimental set-up used to measure the coupling length using the cut-back technique.

The experimental set-up used to measure the coupling length of the fabricated DC mPOF is shown in Figure 3. In order to excite exclusively the fundamental mode of one of the cores of the mPOF at the input end-face, a small light spot of diameter 0.8 µm was focused onto the core of interest by means of a 532 nm laser and a 50× objective. The fiber was mounted on a XYZ linear transition stage so as to align precisely the fiber with respect to the Hamamatsu LEPAS-12 Beam Profiler, based on a CCD camera [39], which included at its entrance a 100× microscope objective that allowed for measurement of the near field pattern at the output end-face of the mPOF. On the XYZ stage, two fiber holders kept the fiber segment closest to the output end-face straight. This fiber segment was cleaved and its coupling length measured by means of the cut-back method. From the output end-face of the fiber, sections of 2 mm in length were cleaved successively. A ruled platform was used to control exactly the cleaving section. Each time the fiber was cut from its output end, the corresponding near-field pattern was measured in order to obtain the power distribution between the cores. 

#### 2.5.2. Selective Excitation of the mPOF

**Figure 4:**
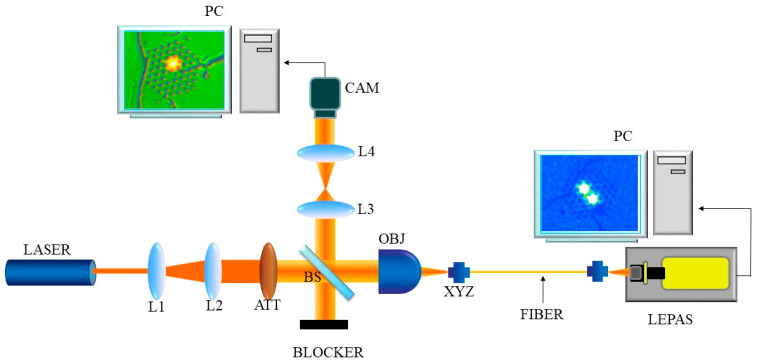
Schematic diagram of the experimental set-up used to excite selectively the fabricated mPOFs. L1: plane-concave lens; L2: symmetrical convex lens; ATT: attenuator; L3: symmetrical convex lens; L4: symmetrical convex lens; BS: beam splitter; OBJ: 0.4–NA objective lens; CAM: digital camera; XYZ: *xyz* linear translation stage.

In order to assess the symmetry of the mPOF, we used the experimental set-up shown in Figure 4. The He–Ne laser beam was expanded and collimated using lenses L1 and L2. The attenuator was aimed at avoiding the saturation of the CCD camera used to monitor the area of the input end-face that was illuminated. The imaging system consisted of the aforementioned camera and imaging lenses L3 and L4, so that the fiber input was viewed magnified in a screen connected to the camera. The exact illuminated position of the end-face was controlled by means of an XYZ stage that allowed a precise positioning of the fiber input. Finally, the objective lens (of numerical aperture 0.4) condensed the collimated light into a spot size sufficiently small to guarantee the selective excitation of individual cores (in Figure 4, see the detail of the screen connected to the camera). 

The near field pattern of five mPOF samples of 38.0 cm in length were measured at two different wavelengths: 594.0 and 612.0 nm. For each fiber sample and wavelength, each of the cores was selectively excited and the corresponding near-field pattern measured at the output end-face. In order to designate unambiguously each core at both ends of the fiber, and thus find the exact correspondence between input cores and output cores, it was very helpful to have a small defect in the form of a small hole created as a consequence of the imperfect fusion of the sleeve.

## 3. Results and Discussion

This section begins by presenting the most relevant numerical simulations that explain the determination of parameters used for assessing the quality of the DC mPOFs fabrication process. The simulation results also support the most representative experimental results that come afterward.

### 3.1. Theoretical Results

Considering *n_co_* = 1.4937 poly (Methyl methacrylate) and making use of the values obtained with the Lumerical software (*n_eff_* = 1.492421 and *n_FSM_* = 1.491239), together with the equations in Section 2.2, the coupling length obtained for the microstructured fiber is 14.6 mm.

### 3.2. Simulation Results

**Figure 5:**
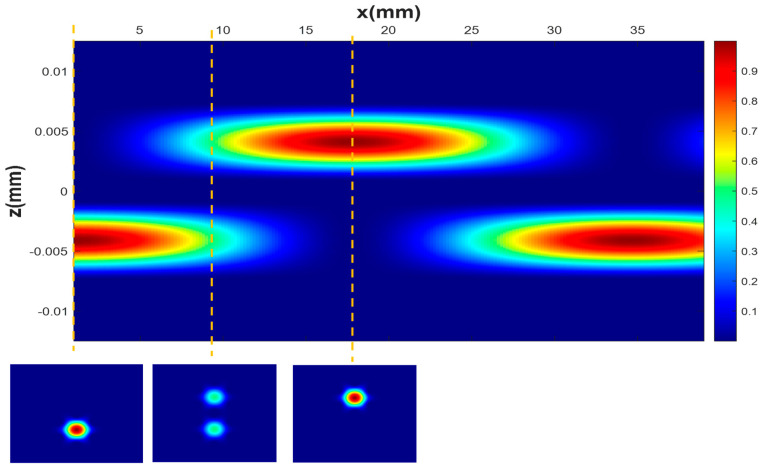
Power oscillations between cores along the longitudinal axis of the mPOF. Figures at the bottom show the near-field patterns at the cross-sections of the mPOF that are marked with dashed lines.

In Figure 5, we can observe the power distribution along the longitudinal axis of the ideal microstructured fiber, where total power transfer between cores is observed. At the input end-face of the fiber, only the lower core is illuminated; however, as light propagates along it, part of the input power couples to the upper core. At z = 8.55 mm, the power is equally distributed between the two cores; whereas, at z = 17.1 mm, all the power is coupled from the lower to upper core. That is, total power transfer occurs and the coupling length is 17.1 mm. This value is similar to that obtained in Section 3.1, although it is not the same. This discrepancy can be attributed to the selected expression for the equivalent core radius (*ρ* = Λ/2), which, although it is the optimal expression for the approach used, is not a perfectly accurate value [35].

**Figure 6:**
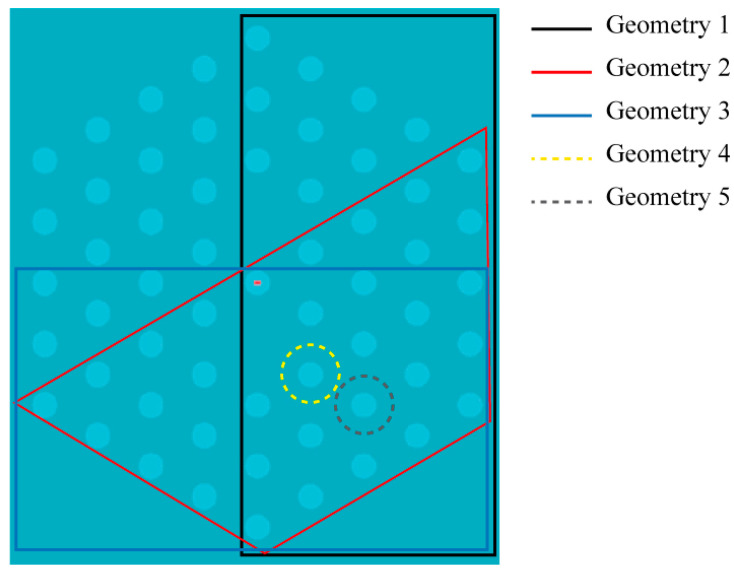
Illustration of the selected regions in the microstructure where geometrical perturbations are applied. The perturbation consists in increasing slightly the size of the holes within the selected region.

Figure 6 illustrates several geometrical irregularities that were created at will in the ideal microstructure, and that result from several simulation instances of interest. More precisely, each simulation instance is obtained from simulating the propagation of light inside the mPOF when a certain set of air holes in the fiber microstructure increased in size with respect to their ideal value (set in Section 2.4). Each selection of holes, to which the perturbation was applied, is specified as Geometries 1 to 5 in Figure 6. Observe that some of the perturbations created concentrate on individual air holes (Geometries 4 and 5), and the rest of geometries on different sets of air holes; Geometry 4 considers a larger air hole within the first ring of one of the cores, whereas Geometry 5 locates the perturbed air hole within the second ring. This approach pursues to simulate situations that may arise because of an inappropriate fabrication process. These geometries are chosen as they can occur as a consequence of a heterogeneous azimuthal heating of the preform in the furnace when drawing (Geometries 1 to 3) or an excessive heating of one of the holes during the drilling process because of a failure in the cooling system (Geometries 4 and 5).

The case of Geometry 1 deserves special attention in the context of power transfer between cores of the perturbed microstructure. It may be noted that the transfer of power continues to be complete as it happens in the ideal case, irrespective of the degree of size increase of the perturbed air holes. Although the microstructure has been modified, this occurs because the solid cores remain symmetrical, that is, the holes surrounding each core are identical for both cores.

**Figure 7:**
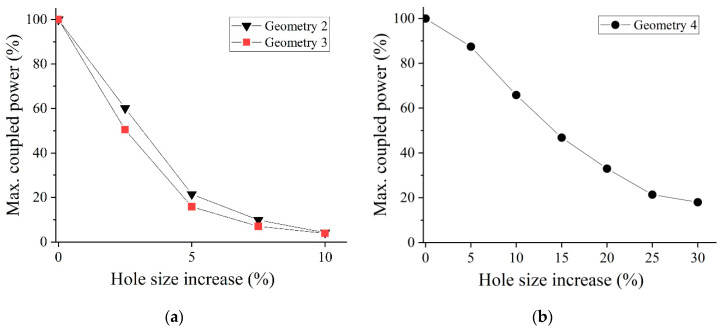
Percentage of coupled power versus radius increase of the selected holes in (**a**) Geometries 2 and 3, and (**b**) Geometry 4.

Figure 7 plots the maximum power transfer between cores as a function of the percentage increase of the perturbed air holes for Geometries 2 to 4. We first concentrate on Geometries 2 and 3. In contrast to what happened in Geometry 1, in these two new cases the symmetry between cores is broken, and as a result of this, the transfer of power is no longer complete. In fact, it is very remarkable the fact that a reasonable increase of 5% in air hole size causes a dramatic decrease in the maximum coupled power, lowering it to values as small as 21.5% in the case of Geometry 2, and 15.8% in Geometry 3. The situation becomes even worse when the percentage increase shifts to 10%; in this case, the maximum coupled power lowers to around 4% in both geometries. Therefore, these results come to confirm that small perturbations in certain sets of air holes have a considerable impact on the power coupling characteristics of the mPOF.

Another instance that may also arise in practice is the effect that a single perturbed air hole would have on the power coupling between the cores (Geometries 4 and 5). If we concentrate on the case of Geometry 4 (Figure 7b), where the perturbed air hole is in direct contact with one of the cores, the size increment of the perturbation continues to cause a decrease in the maximum coupled power, although at a lower rate than in the case of Geometries 2 and 3. When the perturbation size rises to 30%, the maximum coupled power drops to 18.0%, a result that agrees quite well with the 10% maximum coupled power reported in [10]. This accounts for the importance of the perturbations of the microstructure when pursuing a high coupling, as it is in the case of a fiber coupler. Regarding Geometry 5, where the perturbation lies in the second ring of holes surrounding one of the cores, the observed behavior for size increases of 5% and 10%, and is similar to that observed for Geometry 1, i.e., a complete power transfer between cores. Therefore, the fact of having the perturbation located in the second layer (or farther away) has no significant effect on the coupling.

The simulation results included in this section point out that the power coupling characteristic is a key parameter for the assessment of the uniformity of the structure of the fiber, as small disturbances in the structure imply significant power coupling losses, and only when the structure is perfectly uniform can a total power transfer between cores be obtained. Moreover, evaluating the fabrication quality of mPOFs by means of this method has some additional advantages compared to other techniques such as direct imaging. For example, it provides information about the uniformity along the entire fiber segment, and not only about the visible microstructure in the cross-sections.

### 3.3. Experimental Results

**Figure 8:**
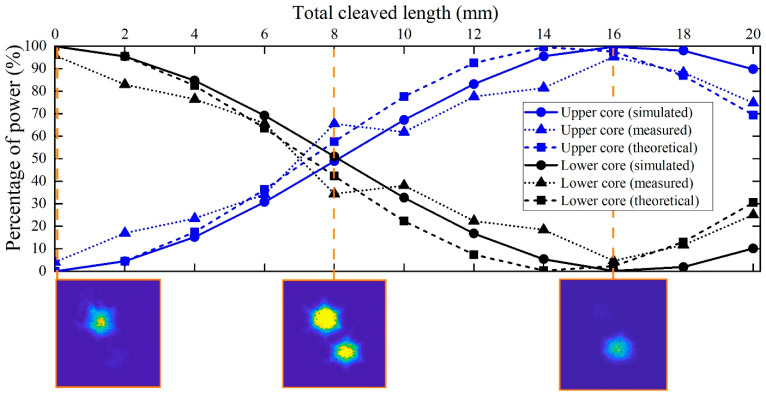
Percentage of power as a function of the total fiber length cleaved from the output end-face of the fiber.

In Figure 8, we start presenting the evolution of the power at each core (as a percentage of the total power) as a function of the total length cut from the output end-face of the fiber. In the same figure, the experimental curve is compared with the theoretical and simulation results. We first draw attention to the theoretical and simulated results. The observed mismatch, which seems to be reasonable, may be attributed to the equivalent core radius (of value Λ/2) chosen in the theoretical model. From Equation (4), we observe that it has a direct impact on the value of the coupling coefficient *C*. If we shift the attention to the experimental curve, it must be first pointed out that the value of the power coupling ratio plotted in the ordinate was derived from the corresponding near-field image using the set-up shown in Figure 3. The photographs below the graph show an example of three near-field patterns measured at three different positions along the longitudinal axis of the mPOF. Each near-field pattern was recorded after cleaving carefully the fiber at the correct position. In each case, the corresponding coupling ratio was obtained from processing the digital image, which allowed for the determination of the number of intensity counts within the core of interest with respect to the total number of counts [39].

Referring back to the previous example shown in Figure 8, the sequence of pictures represents the evolution experienced by the guided light along one coupling length, starting at *z* = 0 where almost all the light is within the lower core (Figure 8), going through *z* = 8 mm in which the upper core already contains 65.6% of the total power, and finally the case at *z* = 16 mm where full transfer of power already occurred from the upper to the lower core. The coupled power curve constructed this way, and shown in Figure 8 as upward-pointing triangles, agrees quite well with the simulated results in average, but the results are not as smooth due to the extremely sensitive measuring procedure. Additionally, the maximum power of 95.94% lies slightly below the maximum expected value of 100%. The experimental error, related to the finite number of cleavings carried out, their quality or the exact excitation of individual cores, is mainly responsible for the observed discrepancy. Finally, to fully appreciate the consequences derived from the experimental results, it must pointed out that the high coupling rate achieved suggests a low geometrical distortion of the microstructure, as we have seen in the numerical simulations—that a certain degree of distortion in the air holes negatively affects the maximum power transfer, pushing its value downward.

**Figure 9:**
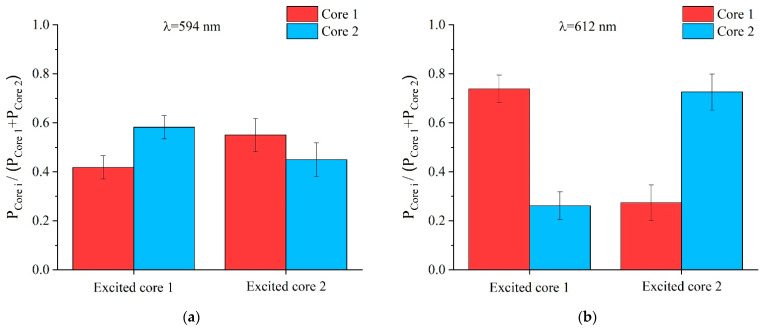
Power ratio (given as average value and standard error of the mean) of each core at the output end-face of a 38 cm-long fiber, when each core is individually excited at wavelengths of (**a**) 594 nm and (**b**) 612 nm (*i* stands for the core index: {1, 2}).

Another aspect that was considered as an additional proof of the fabrication quality was whether the fiber response was completely symmetric to the light excitation. For that purpose, each core was individually excited at two different wavelengths (λ_1_ = 594 nm and λ_2_ = 612 nm) using the set-up shown in Figure 4. The upper row in Table 1 shows the detail of the excitation of one of the cores, and the corresponding near-field patterns at the aforementioned wavelengths. As for the lower row, it shows the same sequence, but when the other core is excited. Note that independently of which core is excited, the power distribution varies with the wavelength of the light excitation. This is a direct consequence of the dependence of the coupling coefficient on the wavelength of light. To acquire a deeper perspective of the results, a bar chart representation of the coupled power at each core is shown in Figure 9. Each bar represents the average value of five measurements, and the corresponding standard error of the mean is plotted as a vertical error bar. At each wavelength, if we take into account the uncertainty associated with the error bars, a clear symmetry of the coupled power with the excited core can be noted. That is, when one of the cores is excited, and the power ratio of each core results in a certain value at the output end; then, if the excitation is moved to the other core, the same value but with interchanged cores is observed.

## 4. Conclusions

To assess the quality of the whole fabrication process of microstructured polymer optical fibers (mPOFs), the optical coupling response of a single-mode (SM) dual-core (DC) mPOF is shown to be a key feature. Numerical simulations carried out on a DC SM mPOF of four rings of holes has shown that the power coupling between cores is especially sensitive to applied geometrical distortions that may arise as a consequence of the fabrication process. It is shown numerically that in most cases of practical interest, the maximum power transfer from one core to the other is well below the expected full power transfer of the undistorted microstructure case.

The experimental results carried out on several samples of mPOFs manufactured in the drawing tower indicate good agreement with the numerical results corresponding to the undistorted microstructure, as a coupling efficiency up to 95.26% was reached. To the best of our knowledge, this is the highest efficiency obtained with an mPOF of such characteristics, which highlights the quality of the fabrication process. Additionally, the coupling length obtained using the cut-back method, as well as the symmetrical response of the mPOF in terms of power distribution between cores at the output end when each core is excited individually, support the numerically predicted expectation for the undistorted microstructure, and ultimately yields useful information about the fabrication process and the almost complete absence of distortions in the downscaling of the preform to the final fiber. This fact reinforces the manufacturing quality and makes this mPOF an appealing alternative to highly efficient power couplers.

## Figures and Tables

**Table 1:** Excited core: images of the fiber cross-section with the laser light exciting each core of the dual-core mPOF; λ = 594 nm: near fields at the output end-face of the fiber for an excitation wavelength of 594 nm; λ = 612 nm: near fields at the output end-face of the fiber for an excitation wavelength of 612 nm.

Excited Core	λ = 594 nm	λ = 612 nm
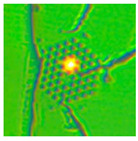	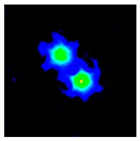	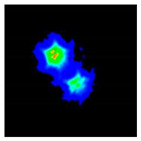
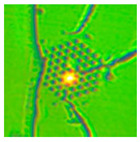	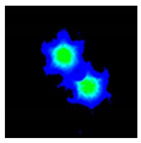	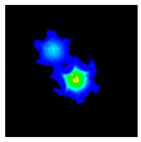

## Data Availability

Not applicable.
